# In Silico, Ex Vivo and In Vivo Studies of Roflumilast as a Potential Antidiarrheal and Antispasmodic agent: Inhibition of the PDE-4 Enzyme and Voltage-gated Ca++ ion Channels

**DOI:** 10.3390/molecules25041008

**Published:** 2020-02-24

**Authors:** Najeeb Ur Rehman, Mohd Nazam Ansari, Abdul Samad

**Affiliations:** 1 Department of Pharmacology & Toxicology, College of Pharmacy, Prince Sattam Bin Abdulaziz University, Al-Kharj 11942, Saudi Arabia; 2 Department of Pharmaceutical Chemistry, Faculty of Pharmacy, Tishk International University, Erbil 44001, Kurdistan, Iraq; abdul.samad@tiu.edu.iq

**Keywords:** antispasmodic, autoDock vina, Ca++ channel blocker, PDE inhibitor, roflumilast, molecular docking

## Abstract

The aim of the present study was to evaluate the possible gut inhibitory role of the phosphodiesterase (PDE) inhibitor roflumilast. Increasing doses of roflumilast were tested against castor oil-induced diarrhea in mice, whereas the pharmacodynamics of the same effect was determined in isolated rabbit jejunum tissues. For in silico analysis, the identified PDE protein was docked with roflumilast and papaverine using the Autodock vina program from the PyRx virtual screening tool. Roflumilast protected against diarrhea significantly at 0.5 and 1.5 mg/kg doses, with 40% and 80% protection. Ex vivo findings from jejunum tissues show that roflumilast possesses an antispasmodic effect by inhibiting spontaneous contractions in a concentration-dependent manner. Roflumilast reversed carbachol (CCh, 1 µM)-mediated and potassium (K+, 80 mM)-mediated contractile responses with comparable efficacies but different potencies. The observed potency against K+ was significantly higher in comparison to CCh, similar to verapamil. Experiments were extended to further confirm the inhibitory effect on Ca++ channels. Interestingly, roflumilast deflected Ca++ concentration–response curves (CRCs) to the right with suppression of the maximum peak at both tested doses (0.001-0.003 mg/mL), similar to verapamil. The PDE-inhibitory effect was authenticated when pre-incubation of jejunum tissues with roflumilast (0.03-0.1 mg/mL) produced a leftward deflection of isoprenaline-mediated inhibitory CRCs and increased the tissue level of cAMP, similar to papaverine. This idea was further strengthened by molecular docking studies, where roflumilast exhibited a better binding affinity (-9.4 kcal/mol) with the PDE protein than the standard papaverine (-8.3 kcal/mol). In conclusion, inhibition of Ca++ channels and the PDE-4 enzyme explains the pharmacodynamics of the gut inhibitory effect of roflumilast.

## 1. Introduction

Gastrointestinal (GI) motility plays an important role in the digestive and absorptive processes of the gut, essential for pushing intestinal material, mixing it with digestive juices, and preparing undigested foods for excretion. Irritable bowel syndrome (IBS) is a gastrointestinal disease related to intestinal motility problems and can be associated with pain, constipation, or diarrhea [1]. Diarrhea, characterized by an increased frequency of bowel movements, wet stool, and abdominal cramps, is a serious health problem [2]. Diarrhea can be caused by several factors, such as infections, food intolerance, intestinal disorders, etc., [3,4,5] and might be caused as a symptom of many other ailments, including IBS, diabetes, etc. [6,7].

Currently available drug treatment for diarrhea is non-specific, and generally, drugs are used to reduce the uneasiness and discomfort of recurrent bowel movements [8]. Available antidiarrheal drugs, such as loperamide used to reduce motility, may prevent diarrhea, and antispasmodic drugs diminish intestinal contractions and decrease the pain [9,10]. Antimuscarinic and other antispasmodic drugs are a valuable therapy in IBS because the smooth muscle relaxant properties of these drugs reduce intestinal spasms [9,10].

The cyclic nucleotides (cAMP and cGMP) act as intercellular second messengers by stimulating the role of many hormones, neurotransmitters, and inflammatory mediators [11,12]. These messengers play an important role in various physiological pathways, including inflammation, immune response, gastrointestinal motility, and reproduction [13]. The phosphodiesterase (PDE) family of enzymes is responsible for the breakdown of these nucleotides, and therefore plays a crucial role in regulating intracellular levels of second messengers and thereby regulating cell physiology [14]. Hence, PDE enzyme inhibition could be one of the potential approaches in treating diseases associated with gut motility [15]. 

Currently, the main focus of researchers is to target one specific PDE enzyme and find out various specific PDE enzyme inhibitors [16]. The selective PDE-4 enzyme is predominant in various inflammatory cells including eosinophils, neutrophils, macrophages, T cells, and monocytes, and it exert its pro-inflammatory effect by degrading cAMP [17]. Previous literature reported that PDE-4 inhibition results in increasing the intracellular cAMP levels and thus initiating various intracellular signaling pathways [18]. PDE-4 inhibitors could be one of the alternative therapies for the management of IBS, since in clinical trials, one of the PDE-4 inhibitors, tetomilast, has shown some benefit for the treatment of inflammatory bowel disease [15].

Roflumilast, a known PDE4 inhibitor, has been known to reduce inflammatory reactions by activating cAMP/PKA pathways [19]. In our previous studies, we reported the cardio and nephron-protective effects of roflumilast [20,21]. However, there is no detailed study available explaining the mechanism of its antidiarrheal and antispasmodic effects. Therefore, in the present study, the antidiarrheal and antispasmodic activities of roflumilast were investigated.

## 2. Results

### 2.1. Effect on Castor Oil-Provoked Diarrhea 

Both orally administered doses of roflumilast showed dose-dependent protection of mice, whereas the saline group did not show any effect. At the lower tested dose of roflumilast (0.5 mg/kg), two out of five mice showed protection, thus showing 40% protection, while the higher dose of 1.5 mg/kg showed 80% protection. No diarrheal spot was observed in any cage of mice treated with loperamide (100% protection), as detailed in Table 1.

### 2.2. Effect on Spontaneous Contractions

**Figure 1:**
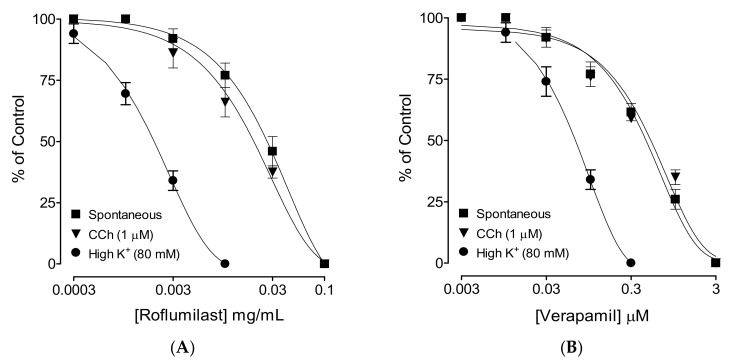
Concentration–response curves showing comparison of (**A**) roflumilast and (**B**) verapamil for their inhibitory effect against spontaneous, carbachol (CCh, 1 µM)- and high K+ (80 mM)-induced contractions in isolated rabbit jejunum preparations. Values shown are mean ± SEM, n = 4–5.

When tested against spontaneously contracting rabbit jejunum preparations, roflumilast caused dose-dependent (0.001–0.1 mg/mL) inhibition with a resultant EC50 value of 0.06 mg/mL (0.04–0.07, 95% CI, n = 4), as shown in Figure 1A. Similarly, verapamil also inhibited spontaneous contractions with an EC50 value of 1.12 µM (0.98–1.68, 95% CI, n = 4) (Figure 1B). 

### 2.3. Effect on Ca++ Curves 

**Figure 2:**
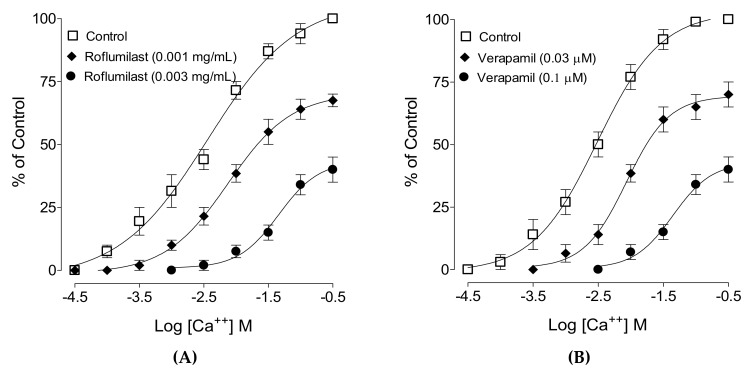
Concentration–response curves of Ca++ in the absence and presence of increasing concentrations of (**A**) roflumilast and (**B**) verapamil in isolated rabbit jejunum preparations. Values shown are mean ± SEM, n = 4–5.

When tested for possible interaction with Ca++ channels, roflumilast was tested against high K+-induced contractions where it produced complete inhibition, similar to verapamil, with respective EC50 values of 0.002 mg/mL (0.001–0.003, 95% CI, n = 5) and 0.1 µM (0.09–0.22, 95% CI, n = 5), as shown in Figure 2. To further confirm the Ca++-inhibitory effect, roflumilast-pretreated jejunal preparations with doses of 0.001 and 0.003 mg/mL produced a rightward shift in the Ca++ curves (Figure 2A), similar to that caused by verapamil (Figure 2B). 

### 2.4. PDE-Inhibitory Effect

**Figure 3:**
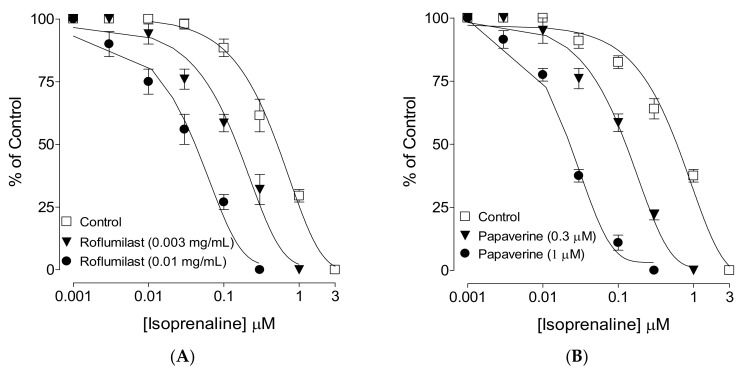
Inhibitory concentration–response curves of isoprenaline against carbachol (CCh)-induced contractions in the absence and presence of different concentrations of (**A**) roflumilast and (**B**) papaverine, in isolated rabbit jejunum preparations. Values shown are mean ± SEM, n = 4-5.

When tested against CCh-induced contractions, roflumilast produced dose-dependent (0.001-0.1 mg/mL) inhibition with a resultant EC50 value of 0.07 mg/mL (0.05–0.08, 95% CI, n = 4), as shown in Figure 1A. Pretreatment of tissues with roflumilast (0.03 and 0.1 mg/mL) shifted the isoprenaline-induced inhibitory concentration–response curves (CRCs) to the left (Figure 3A), showing a potentiating effect. Papaverine (0.3–1 μM) also caused a similar leftward shift of the isoprenaline curves, as shown in Figure 3B. 

**Figure 4:**
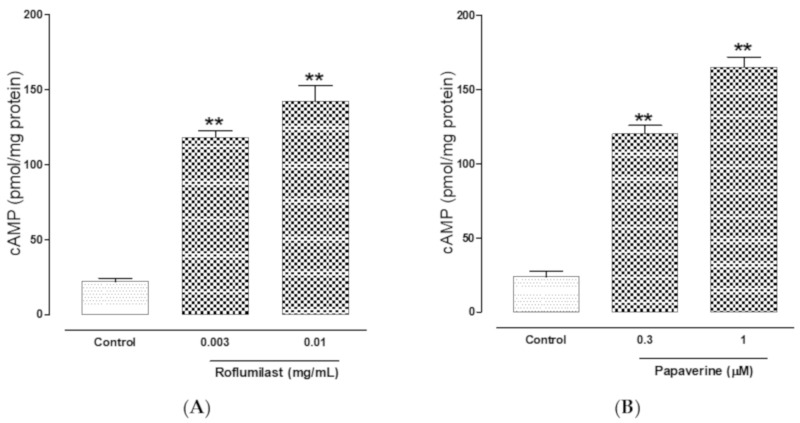
Effect of roflumilast (**A**) and papaverine (**B**) on the cAMP content of rabbit jejunum.

The PDE inhibitory effect of roflumilast also confirm by estimating cAMP levels in tissues by biochemical method. The cAMP levels of untreated tissues’ homogenates were measured 22.52 ± 2.15 pmol of cAMP/mg protein compared to roflumilast pre-incubated tissues with increasing concentrations of 0.003 and 0.01 mg/mL where respective concentrations of cAMP measured were 118.16 ± 4.5 (*p* < 0.01) and 142.71 ± 10.4 pmol/mL (p < 0.01) (Figure 4A). Papaverine pretreated jejunal tissues also caused increase in the levels of cAMP up to 120.07 ± 5.64 (*p* < 0.01) and 165.93 ± 6.80 pmol/mL (*p* < 0.01), at respective doses of 0.3 and 1µM **(**Figure 4B). 

### 2.5. Molecular Docking Analyses

**Figure 5:**
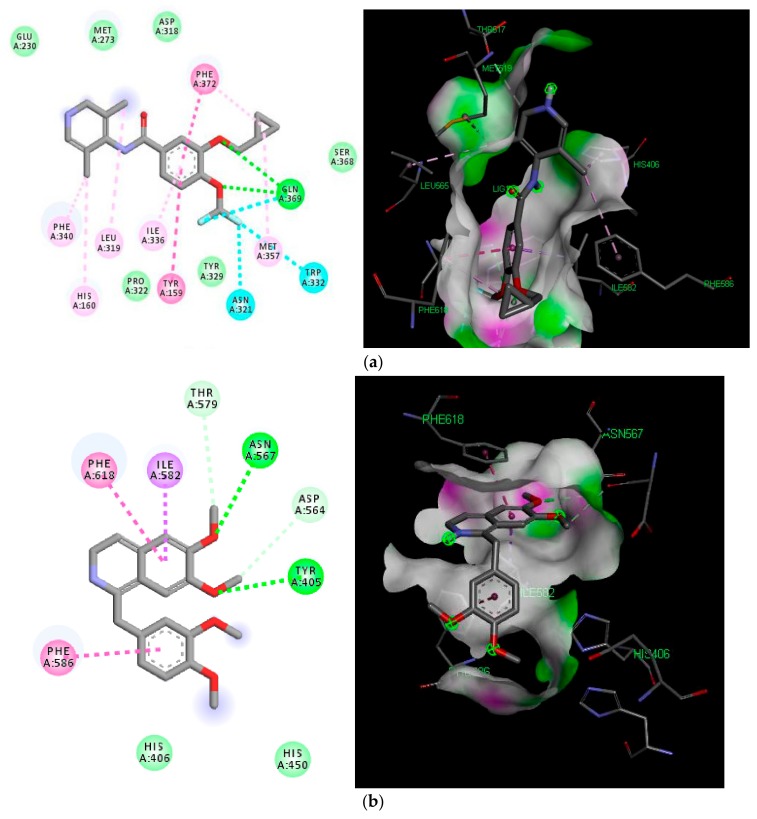
(**A**) 2D and 3D diagrams of the docked structure of roflumilast in the active domain of PDE-4 (5LAQ). (**B**)**.** 2D and 3D diagrams of the docked structure of papaverine in the active domain of PDE-4 (5LAQ). Green dashed lines represent hydrogen bonding, cyan lines represent halogen bonding, light purple lines represent Sigma–Pi interaction.

Furthermore, to understand the PDE-4 inhibition, roflumilast and papaverine were docked into the active pockets of PDE-4B and PDE-4D proteins having PDB ID 5WH5 and 5LAQ, respectively. Roflumilast exhibited better binding affinity (−9.4 and −9.3 Kcal/mol) in comparison to papaverine (−8.3 and −8.2 Kcal/mol) in both the isoforms, as shown in Table 2. Roflumilast made two significant hydrogen bonds with Gln615 and Asn567, and halogen bonds with Gln615 and Trp576 while papaverine interacted with the protein by two distinct hydrogen bonds with Tyr405 and Asn567, as shown in Figure 5A,B, respectively. The notable difference in the binding affinities of roflumilast and papaverine can be attributed to the hydrogen–halogen bonding of the former. The hydrogen bond apart from the halogen bond in roflumilast represents an interaction of halogen and hydrogen bond donors on the side chain of the PDE protein, which is considered substantially more favorable in comparison to a canonical hydrogen bond.

## 3. Discussion

In view of the wide therapeutic potential of roflumilast, we previously reported studies showing its effectiveness in the treatment of inflammatory conditions and related diseases such as cardio and renal toxicities [20,21]. The current study was therefore planned to test the additional possible therapeutic potential of roflumilast in hyperactive disease states of the gut in mice, with detailed investigation of the mechanism(s) using rabbit jejunum in ex vivo experiments. Induction of diarrhea in normal mice was made possible using castor oil, which after hydrolysis into ricinoleic acid, led to the evocation of spasms in the gut [22]. Roflumilast pre-administration protected mice from diarrhea in a dose-mediated way, similar to the positive control drug, loperamide, a typical antidiarrheal agent [23]. 

After we observed the antidiarrheal response, our second objective was to determine the possible pharmacodynamics involved in this effect. For this purpose, the method of Bashir et al. [24] was followed by testing cumulative concentrations of roflumilast in an isolated, spontaneously contracting jejunum. Interestingly, roflumilast exhibited complete inhibition, thus showing an antispasmodic effect and further supporting our in vivo observations. Based on our previous observations that antispasmodic agents mediate gut inhibitory effects by blockade of Ca++ channels [23], potassium channel activation [25] and/or PDE inhibition [26], we further tested roflumilast on provoked contractions in rabbit jejunum with the respective contractile agents of high K+, low K+ and carbachol [27]. Critical analysis of the patterns of the inhibitory CRCs of roflumilast against high K+ and CCh shows that it exhibited statistically higher potency against high K+ in comparison to CCh, a typical property of Ca++ channel blockers (CCBs) [28]. Our positive control drug, verapamil, a known voltage-gated Ca++ inhibitor [29], also reversed high K+ with higher potency than CCh, thus further strengthening our findings. No significant difference was observed in the potency of roflumilast against high K+ and low K+ (data not shown), thus ruling out the possible role of K+ channel activation as one of the antispasmodic mechanisms of roflumilast. A test sample that selectively reverses low K+ (<30 mM)-mediated spasms depicts a K+ channel opener, while CCBs relax both low and high K+-mediated spasms with equal potencies, hence these investigations differentiate K+ channel openers from CCBs [30,31]. To further support and confirm the CCB-like action, roflumilast was pre-incubated in jejunal tissues stabilized in Ca++-free buffer solution. Contractile CRCs of Ca++ were measured in jejunal tissues in the absence and presence of pre-incubation with increasing doses of roflumilast. Roflumilast deflected Ca++ CRCs to the right with suppression of the maximum peak, similar to verapamil, thus confirming its CCB-like effect. Roflumilast, being known for PDE inhibition [19], was tested indirectly for PDE inhibition and elevation in the cAMP levels as PDE inhibitors, by blocking PDE, cause an elevation in tissue cAMP levels. PDE is responsible for converting cAMP to its inactive form (AMP), thus resisting smooth muscle relaxation [32]. Roflumilast, however, completely reversed CCh-mediated contractions with more potency compared to its effect against high K+, thus pointing toward PDE inhibition as an additional gut inhibitory mechanism of roflumilast. It is well known that PDE inhibitors inhibit CCh-mediated spasms of smooth muscles [33]. The potentiation of the inhibitory CRCs of isoprenaline towards lower doses (leftward) in jejunal tissues pre-incubated with roflumilast indirectly confirms its PDE-inhibitory character which was further confirmed when roflumilast pre-incubation causes increase in the levels of cAMP in jejunal tissues, similar to papaverine, a standard PDE inhibitor [34,35]. 

The PDE inhibition concept was further proved concretely by the in silico molecular docking results. The molecular docking study helped us to gain an insight into the protein–ligand interaction in the active domain of PDE-4. PDE-4 is reported to have four subtypes (A, B, C and D) each with the potential to degrade cAMP, which is the second messenger to react to the inflammatory effect. Roflumilast is a selective and non-specific PDE4 inhibitor that is able inhibit all PDE-4 isoforms. Despite its pharmacological effects, roflumilast can cause side-effects, such as diarrhea, nausea and dizziness, due to a lack of specificity. As PDE-4B and PFDE-4D have been found to be more expressed and extensively studied, hence were selected for the present docking study. The standard drug papaverine exhibited two hydrogen bonding affinities, while roflumilast exhibited better binding affinity with two hydrogen bond and two hydrogen–halogen bonds. Halogen and hydrogen–halogen bonding has been considered more favorable, and this is why halogen-containing compounds are being further explored and believed to be dual agonists [36].

## 4. Materials and Methods 

### 4.1. Chemicals and Reagents

The following analytical-grade chemicals, obtained from Sigma (USA), were used: carbamylcholine (CCh), loperamide, acetylcholine perchlorate (ACh), isoprenaline, verapamil, and papaverine. Castor oil was purchased from a local pharmacy. 

### 4.2. Animals 

Rabbits (1-1.5 kg) procured from a local farm in Al-Kharj and Swiss albino mice (20-25 g) from the Animal Care Unit, College of Pharmacy, Prince Sattam bin Abdulaziz University, Saudi Arabia, were kept at optimum temperature (22 ± 1 °C), relative humidity 55% ± 5%, and equal exposure to light/dark cycle. All animals were provided with a standard pellet diet and water ad libitum. Before sacrifice, rabbits fasted for 24 h. Cervical dislocation was performed by a blow on the neck, and death was confirmed by the disappearance of ear reflexes. Due care was taken, and all experiments (in vivo and ex vivo) were performed by following the instructions detailed in NRC [37].

### 4.3. In Vivo Experiments 

Twenty mice were randomly allocated into four groups with equal numbers in each group. Following twenty-four-hour fasting, mice in the 1st group were exposed to oral gavage of saline (10 mL/kg) and were labeled as a negative control. After pilot screening for dose selection, the 2nd and 3rd groups (test groups) were exposed to two increasing dosages of 0.5 and 1.5 mg/kg of roflumilast, respectively. The last group (4th) of mice were given loperamide (10 mg/kg) and labeled as a positive control. Separate cages were assigned to each animal, with a blotting sheet on the floor of each cage for observation of the absence or presence of diarrhea by a blind observer. After an hour, all mice were orally exposed to castor oil (10 mL/kg) using a 1 mL syringe. After 4 h, all individual cages’ blotting sheets were inspected for typical diarrheal droppings. Protection was noted if no diarrheal drops were observed, as previously reported by Jebunnessa et al. (2009) [38]. 

### 4.4. Ex Vivo Experiments 

#### 4.4.1. Antispasmodic Effect 

The method of Shah et al. (2011) [39] was followed to sacrifice the rabbit and to isolate the middle part of the small intestine (jejunum). Following isolation, the required segments of jejunum 2–3 cm in length were cleaned of any adjacent tissues and fecal material, and were mounted in an emkaBath (France) attached with a transducer and IOX software. Fresh Tyrode’s solution was filled in 20 mL tissue baths gassed with carbogen, and the temperature was set at 37 °C. The composition of Tyrode’s solution was followed as previously described by Khan et al. (2018) [40]. Tension of 1 g was applied by rotating the transducer knob clockwise, and the tissues were left for stabilization for 30 min with multiple exposures of acetylcholine (0.3 µM). After spontaneous contractions of the jejunum were found with a stable band, roflumilast was added to the bath solution in increasing concentrations. 

After observing the inhibitory effect of roflumilast on spontaneous contractions, experiments were further extended to determine the mechanism(s) of its antispasmodic effect. The inhibitory effect of roflumilast was tested against sustained spasms mediated by K+ (80 mM) and CCh (1 µM) for multiple pharmacodynamics including voltage-gated Ca++ channel blockade and inhibition of PDE. According to Godfraind et al. (1986) [41], K+ (>30 mM) depolarizes multiple smooth muscles by causing activation of Ca++ channels (L-type), thus causing sustained contractions. Similarly, any drug that reverses CCh-mediated contractions is considered as antimuscarinic and/or a PDE inhibitor [42,43].

#### 4.4.2. Ca++ Channel-Blocking Activity

Following the preliminary inhibitory effect of roflumilast against high K+, confirmation of Ca++ channel blockade was determined by 30 min incubation of jejunal tissues in Ca++-free Tyrode’s solution with EDTA (0.1 mM) for Ca++ chelation. To further deplete the intracellular stores of Ca++, the method of Blattner et al. (1978) was followed [44]. Briefly, Ca++-free solution was exchanged with another solution labeled as K+-rich and Ca++-free Tyrode’s solution with the following concentrations (mM): potassium chloride, 50; sodium chloride, 91.03; sodium dihydrogen phosphate dehydrate, 0.32; sodium bicarbonate, 11.9; magnesium chloride hexahydrate, 0.50; glucose, 5.05; and calcium chelating agent (EDTA), 0.1. At the end of 1 h of preservation of jejunal tissue in this solution, Ca++ was added exogenously to produce dose-dependent CRCs in the absence and presence of two increasing doses of roflumilast. Parallel experiments were run using verapamil as a positive control. 

#### 4.4.3. PDE-Inhibitory Activity

As roflumilast showed efficacy against CCh, which is an indication of PDE inhibition, indirect confirmation was carried out by dose-mediated inhibitory curves of isoprenaline against CCh in the absence and presence of roflumilast. Potentiation of curves to the left is indicative of a blockade of PDE, while papaverine, a standard PDE inhibitor, was used for comparison [45].

Further confirmation of PDE inhibitory activity was done by biochemical method using cAMP enzyme immunoassay kit (ARBOR assays Detect X, direct cAMP enzyme immunoassay kit, Ann Arbor, MI, USA). Jejunal tissues, after contracting with CCh (1 µM), were exposed to relaxant doses of roflumilast and papaverine. Soon after the tissues reached to baseline, the tissues were snapped frozen in liquid nitrogen and were homogenized with 1 mL of sample diluent for every 100 mg of tissue, on ice, which was kept like this for 10 min. After centrifugation at ≥6000 g (4 °C for 15 min), the supernatant was collected and either analyzed immediately. Similar steps were repeated for tissues without administration of any drug and only contracted with CCh, which served as negative control. The cAMP content was expressed as picomole per mg protein.

### 4.5. In Silico Studies

To gain an insight into a PDE protein and its interaction with roflumilast, co-crystallized ligand and papaverine, molecular docking studies were carried out on two isoforms, namely PDE-4B and PDE-4D, using the Autodock vina program from the PyRx virtual screening tool [46,47]. The crystal structure of the PDE protein was downloaded from the protein data bank with PDB ID: 5LAQ and 5WH5 (resolution 2.4A and 1.4A, respectively), and it was further subjected to repairing and extraction of the co-crystallized ligand using Discovery studio visualizer (DSV) 4 [48]. The co-crystallized ligands, were redocked into the active domain of their respective proteins to validate the docking methodology used in the study. The 3D structure of roflumilast was downloaded from PubChem and with the help of open babel, it was minimized and converted to pdb format. DSV was used for visualization of the docked poses. 

### 4.6. Statistical Analysis 

Data are expressed as the mean ± standard error of the mean (SEM), where n is the number of experiments and EC50 represents the median effective concentration with 95% confidence interval (CI). Protection from diarrhea was statistically evaluated by comparing all groups with the saline control group using Chi-square (x^2^) test. Statistical significance was considered only when *p* < 0.05. Graph Pad Prism (version 4) was used for the analysis of concentration–response curves (CRCs).

## 5. Conclusions

These findings show that roflumilast possesses antidiarrheal and antispasmodic effects, possibly mediated by blockade of voltage-gated Ca++ channels and PDE inhibition with resultant increase in cAMP levels, although the possibility of β2-adrenergic receptor and/or adenylyl cyclase activation mechanisms cannot be ignored.

## Figures and Tables

**Table 1:** Antidiarrheal activity of roflumilast on castor oil (10 mL/kg)-induced diarrhea in mice.

Treatment (p.o.)	No. of Mice with Diarrhea	% Protection
Saline (10 mL/kg) + Castor oil	5/5	0
Roflumilast (0.5 mg/kg) + Castor oil	3*/5	40
Roflumilast (1.5 mg/kg) + Castor oil	1*/5	80
Loperamide (10 mg/kg) + Castor oil	0**/5	100

* *P* < 0.05 and **P < 0.01 vs. Saline + Castor oil-treated group (x^2^-test).

**Table 2:** Molecular docking scores with PDE-4B (5WH5) and PDE-4D (5LAQ) isoforms.

Name of Molecule	Binding Energy (Kcal/mol)			Types of Bonding
	5WH5	5LAQ	5WH5	5LAQ
Roflumilast	−9.4	−9.3	H-bond, Halogen bond, Van der Waals, Pi-sigma, Pi-Pi.	H-bond, Halogen bond, Van der Waals, Pi-sigma, Pi-Pi,
Papaverine	−8.3	−8.2	Van der Waals, Pi-sigma, Pi-Pi	H-bond, Van der Waals, Pi-sigma, Pi-Pi,
Co-crystallized ligand	−10.6	−8.6	H-bond, Halogen bond, Pi-sigma, Pi-Pi	H-bond, Pi-Cation, Pi-sigma, Pi-Pi,
